# Supervised Machine Learning to Identify Hospital Inpatients Needing a Change of Antibiotic Therapy in Real Time: Preclinical Diagnostic Evaluation and Feasibility Study

**DOI:** 10.1093/ofid/ofaf721

**Published:** 2025-11-25

**Authors:** P F Dutey-Magni, M Brown, S Harris, C Curtis, R Dobson, H Chowdhury, A Cawthorn, S De, N Stone, J Cooper, L Shallcross

**Affiliations:** Institute of Clinical Trials and Methodology, University College London, London, United Kingdom; Infection Department, University College London Hospitals NHS Foundation Trust, London, United Kingdom; Department of Clinical Research, London School of Hygiene & Tropical Medicine, London, United Kingdom; Biomedical Research Centre, University College London Hospitals NHS Foundation Trust, London, United Kingdom; Institute of Health Informatics, University College London, London, United Kingdom; Department of Infection Sciences, King's College London NHS Foundation Trust, London, United Kingdom; Institute of Health Informatics, University College London, London, United Kingdom; Infection Department, University College London Hospitals NHS Foundation Trust, London, United Kingdom; Advanced Research Computing Centre, University College London, London, United Kingdom; Department of Microbiology, Virology and Infection Control, Great Ormond Street Hospital for Children NHS Foundation Trust, London, United Kingdom; Infection Department, University College London Hospitals NHS Foundation Trust, London, United Kingdom; Centre for Clinical Microbiology, University College London, London, United Kingdom; Advanced Research Computing Centre, University College London, London, United Kingdom; Institute of Health Informatics, University College London, London, United Kingdom

**Keywords:** antimicrobial stewardship, infectious disease medicine, medication review, secondary care, supervised machine learning

## Abstract

**Background:**

Postprescription review (PPR) by clinical microbiology/infectious diseases specialists is a proven intervention for optimizing antibiotic management in hospitals. However, hospitals lack sufficient staff to conduct PPR at scale. This study investigated the feasibility and diagnostic performance of supervised machine learning to monitor electronic medical records and prioritize PPR in real time.

**Methods:**

Over 2 years, infection specialists categorized PPR recommendations as recommendations to “stop,” “change,” or “continue” therapy at an acute hospital in London, United Kingdom. These labels (n = 2625) were linked to features generated from electronic patient records. Random forest, XGBoost, and C5 classifiers were trained before undergoing prospective evaluation in an unseen representative validation dataset (446 PPR decisions). The prespecified minimum predictive performance target was an area under the curve (AUC) of 0.75, with statistical power to detect an AUC <0.68 or >0.82. We then aggregated the validation dataset at the patient level (n = 358) and compared the clinical utility of targeted PPR using the classifier against unprioritized (random) PPR.

**Results:**

In the prospective validation set, 145 of 358 patients (41%) were recommended to change or stop treatment. The best-fitting classifier (random forest, cross-validation AUC, 0.74) achieved an AUC of 0.70 (95% confidence interval, .65–.75). If just the top 30% of patients receiving antibiotics could be reviewed, the classifier would help stop or change treatment in 68 of 145 patients requiring a change, compared with 43 of 145 if patients were selected at random.

**Conclusions:**

The prospective clinical evaluation demonstrated that the approach is feasible and achieves moderate predictive performance in real-world conditions.

## Teaser

This study evaluated the diagnostic performance of a supervised machine learning classifier prioritizing hospital inpatients for antibiotic therapy review. It found moderate predictive accuracy and clinical utility to enhance antimicrobial stewardship in hospitals equipped with electronic health records.

In 2019, drug-resistant infections claimed 1.3 million lives globally [1], and this number could escalate to 10 million by 2050 [2]. A crucial factor in the rise of drug-resistant pathogens is the overuse of antibiotics in healthcare [3]. One in 3 hospital inpatients is receiving antibiotics at any time [4], while audits reveal that 30%–50% of prescriptions are either inappropriate or not compliant with antibiotic guidelines [5, 6].

A range of antimicrobial stewardship strategies have been proposed, which can reduce antibiotic treatment duration by an average 2 days [7–10] and the risk of certain drug-resistant infections by 50% [11]. One such intervention is postprescription review (PPR) by professionals trained in clinical microbiology/infectious diseases [6, 12, 13], hereafter referred to as “infection specialists.” In a natural experiment conducted in a UK hospital [14], infection specialists were 4 times less likely to initiate antibiotic therapy than other medical consultants and used 30% shorter courses. A multicenter randomized controlled trial found that PPR reduced prescription duration by >3 days [15]. A crossover trial [16] found that PPR with feedback improved antibiotic appropriateness and shortened treatment compared with preprescription authorization, because of more information being available 48 hours after initiation. PPR may be conducted by infection specialists or specialist pharmacists [13, 17].

**Figure 1.**
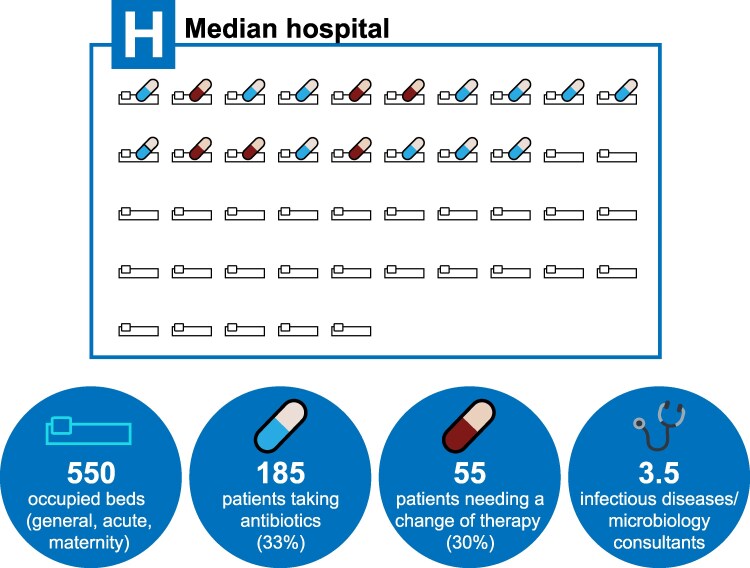
Diagram of the median hospital's occupied beds and inpatients needing a change of antibiotic therapy (based on point prevalence survey and staffing data from Plachouras et al [4], NHS England [18], and NHS Digital [19]).

However, antimicrobial stewardship faces a resource challenge. In England, hospitals care for a median of 550 general/acute/maternity patients at any time, one-third of whom receive antibiotics [18]. Assuming that treatment should be changed or discontinued in 30% of patients [5–7], infection specialists need to identify and change therapy for 55 of 185 patients receiving antibiotics (Figure 1). This exceeds the typical capacity of most teams [20–22].

This challenge could be addressed if patients could be prioritized in such a way that infection specialists review only the patients most likely to need a change of therapy. The present study investigated the feasibility of using a supervised machine learning algorithm to predict the probability that a patient needs antibiotics stopped or changed, using real-time records from electronic prescribing, laboratory, and medical notes systems. If proved feasible and safe, such an algorithm could be incorporated within a software medical device generating prioritized patient lists, allowing infection specialists to maximize the clinical impact of the time dedicated to PPR.

## METHODS

### Study Design

The study consisted of 2 stages. In the first stage, *retrospective training*, a supervised classification model was trained to predict recommendations issued by infection specialists (consultants and trainees) as they reviewed antibiotics after prescription (“stop therapy,” “change therapy,” or “no change”). The model was trained using information available at the time of review from routinely collected electronic hospital records. In the second stage, *prospective validation,* infection specialists at the University College London Hospitals NHS Foundation Trust (UCLH) reviewed a prospective random sample of inpatients taking antibacterial drugs and recorded the decision (stop therapy, change therapy, or no change). This dataset was used to estimate the diagnostic accuracy of the model independently from the training dataset, and look for signs of generalization bias.

The intended purpose of the model was to rank hospital inpatients according to the probability that an infection specialist would recommend stopping or changing an antibiotic prescription. We aimed for the model to help double the number of patients changing therapy. For this, we set an a priori predictive performance target in terms of full area under the receiver operating characteristic curve (AUC). This metric evaluates how well a diagnostic tool ranks subjects by the probability of a specific condition. The AUC ranges from 0.5 (no diagnostic value, equivalent to random ranking) to 1 (perfect diagnostic performance). By simulating a range of binormal distributions, we determined that a minimum AUC performance of 0.7 would likely achieve the increase in yield we were targeting based on a detailed review of the use case reported in the Supplementary Material 7.

These operating characteristics can be translated to a typical hospital scenario to represent the expected clinical utility, as presented in Figure 1. Assuming that 55 of 185 patients (30%) receiving antibiotics need to change or stop therapy, 45% sensitivity means that infection specialists could stop or change therapy for 25 patients (55 × 45%), provided that they review just 36 patients, thanks to a 70% positive predictive value (PPV) (55 × 45%/70%). In comparison, by reviewing a random sample of 36 patients, infection specialists would change therapy in just 11 patients (36 × 30%, based on a 30% prevalence of inappropriate therapy). With those parameters, the yield is changing therapy in an additional 25 patients.

### Setting

The study took place at the UCLH, a tertiary care organization with 539 acute/general/maternity beds in London, United Kingdom. UCLH's main hospital consists of an emergency department, acute medical unit, intensive care unit, and specialty wards (including an infectious diseases ward). The organization's 6 other inpatient hospitals provide more specialist care, including their own specialty critical care units. The organization is equipped with Epic clinical systems for patient administration and electronic medical notes (Epic Systems) and a laboratory information system (WinPath; Clinisys).

The organization includes a team of 18 consultants trained in clinical microbiology and/or infectious diseases, who oversee 25 specialty trainees. The hospital has established regular antibiotic stewardship ward rounds in some medical and surgical specialties (weekly in oncology, neurology/neurosurgery, and urology, daily in infectious diseases). Antibiotic prescribing guidelines are developed locally and available on a web app. For key infections, first-choice empirical therapy recommendations are consistent with national recommendations. Some restricted agents may be ordered only by microbiologists (eg, meropenem, tobramycin, and intravenous vancomycin). Prescribing of other agents outside preapproved indications is also restricted (amikacin, third-generation cephalosporins, intravenous or oral chloramphenicol, piperacillin-tazobactam, and oral vancomycin).

### Participants

For model training, eligible records were those belonging to inpatients aged ≥18 years discharged between April 2019 and March 2022. For the prospective validation dataset, eligible patients were inpatients aged ≥18 years on admission, admitted between November 2021 and April 2022, taking systemic antibacterials during their admission. We excluded prescriptions for long-term therapy or prophylaxis for tuberculosis (rifaximin) and complex opportunistic infections in HIV-infected patients, such as *Pneumocystis* pneumonia, toxoplasmosis, cryptosporidium, and other parasitic infections (dapsone, pyrimethamine, sulfadiazine, and pyrimethamine). Those treatments are typically long term (≥6 months) and overrepresented due to specialist HIV and tuberculosis units hosted in our center.

### Outcome

Outcome data for model training were antibiotic prescription reviews completed as part of routine antibiotic stewardship ward rounds (1) between September 2019 and October 2020 in the neurology/neurosurgery hospital only, using a spreadsheet (n = 621); and (2) between November 2020 and March 2022, across all hospitals/wards, using electronic progress notes extracted and parsed from the hospital data warehouses (n = 2004). When reviewing antibiotics, infection specialists recorded their recommendation (stop, change drug, change dose, lengthen, shorten, intravenous to oral, oral to intravenous, or no change) into a clinical note template (Epic SmartPhrase [23]), which was subsequently reclassified into 3 categories (stop, change, or no change) to train the model. Learning and prediction took place at the prescription level, while validation was conducted at the patient level. For any given patient, the prediction target was the highest value of the individual prescriptions' predicted probabilities of being changed or stopped.

### Features

**Figure 2.**
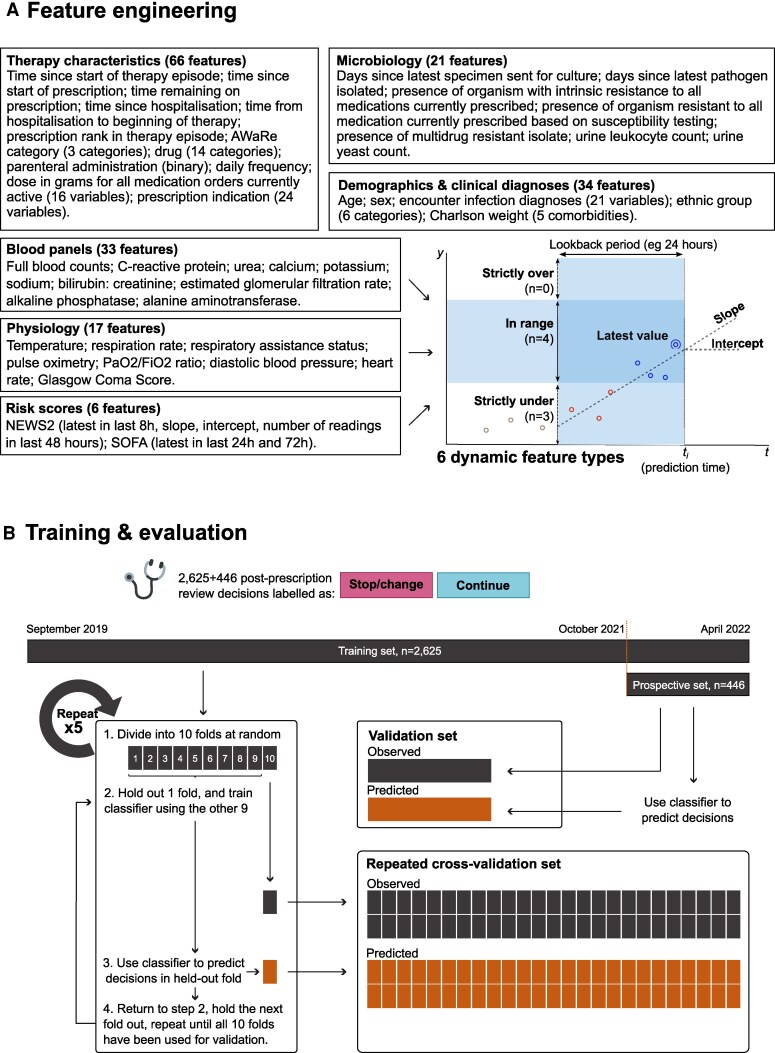
Design overview of the machine learning pipeline: *A*, Overview of learning features. Chart of features *y* against time *t* illustrates 6 dynamic feature types constructed by the Ramses software package: latest value; numbers under, within, or over prespecified range; and slope/intercept. All features require specifying a lookback period. *B*, Overview of labels collected for training and evaluation data, showing the training and prospective evaluation sets and a diagram explaining the repeated cross-validation procedure. Abbreviations: Fio_2_, fraction of inspired oxygen; Pao_2_, partial pressure of arterial oxygen; PPR, postprescription review; SOFA, Sequential Organ Failure Assessment.

Patient administration records, clinical diagnoses, patient physiology, pathology test results, antibacterial medication orders (prescriptions), and administrations records were extracted and pseudonymized for analysis. Data modeling and feature engineering (Figure 2*A*) were handled by the Ramses (version 0.5.2) [24] and AMR (version 1.8.1) packages [25] in R software (version 4.1.3) [26].

Antibacterial medication order records were processed using the Ramses package and linked into “therapy episodes,” defined as the set of antibiotic prescriptions concurrently or successively administered without an interruption lasting >36 hours [24, 27]. The functions of the Ramses package allow the construction of a matrix with 1 row for every hour of the therapy episode, tracking clinical features throughout the period during which a patient is taking antibiotics. Prescription doses were all converted to grams and summarized by antibiotic group (see Figure 2*A* and Supplementary Material 2). Prescription indications (recorded as a combination of buttons and free text) were mapped to SNOMED CT terminology with the snomedizer package [28]. Prescriptions were then coded in relation to 84 infection categories using the SNOMED CT semantic inference, as described in Supplementary Material 1.

Features were also generated from records of microbial specimens sent for culture, pathogens isolated, and antibiotic susceptibility testing results as released by microbiologists (rather than the complete susceptibility profile). Expert rules from the European Committee on Antimicrobial Susceptibility Testing [29] were applied using the AMR package before deriving features. Feature definition and selection is reported in detail in Supplementary Material 2.

The design of features derived from physiology and laboratory data was informed by past research [30–34] and observation of infection specialists as they reviewed antibiotics. Three types of features were generated by the Ramses software package over a predetermined lookback period from the time of prediction (Figure 2*A*): the latest observation available (if any); the number of observations falling strictly under, within, or strictly over a range or threshold; and the ordinary least squares slope and intercept at prediction time. Documentation is available online [24].

### Sample Size

The size of the prospectively sampled validation dataset was set to estimate the clinical utility of the classifier's prediction that prescriptions should be stopped or changed versus continued with no change. The null hypothesis was defined as the AUC meeting a minimal clinical utility of 0.75, with equivalence margins of 0.68–0.82, 80% power, and 95% confidence. Based on formulas from Obuchowski [35], an a priori sample size of 720 was determined sufficient based on a previous clinical audit predicting a ratio (*R*) of “stop” or “change” to “no change” decisions of 5 (ie, 20% of prescriptions), but the protocol provided for an update of parameters based on an internal pilot consisting of the initial 250 observations. This interim review revealed that 83 of 250 prescriptions were recommended for stopping or changing therapy, equivalent to *R* = 2. With this revised parameter, we determined that a final target sample size of 446 prescription review decisions was sufficient to test the hypothesis.

### Statistical Modeling

We investigated 3 supervised classifiers: random forest [36], XGBoost [37], and C5 [38]. Classifiers were tuned on accuracy and AUCs were measured using repeated 10-fold cross-validation in the full training dataset. The following measures of importance were examined: normalized permutation importance (random forest), gain (XGBoost), and usage (C5). The prevalence of missing values in training features is reported in Supplementary Material 2. XGBoost and C5 were configured to build missingness within decisions trees. Random forest models were imputed with the training dataset medians, unless otherwise stated in the Supplementary Materials.

### Evaluation

The diagnostic performance of all classifiers was evaluated independently in the prospective validation dataset at prescription level, to measure the binary AUC of predicting stop/change versus no change decisions and test the hypothesis that the AUC was equivalent to 0.75, using a DeLong test [39] at a .05 significance threshold. We also produced calibration curves on the binary prediction task (comparing model probabilities with observed event rates over 5 splits) and computed the Brier score.

Clinical utility was also evaluated in the same dataset, this time at the patient level (presence of ≥1 decision to change or stop antibiotic therapy). For this, the best classifier was used to rank patients by the maximum predicted probability of stop/change decisions in all active prescriptions and identify the top 10%, 20%, and 30% of patients to simulate an algorithm-driven ward round. Of those 10%, 20%, and 30%, we measured the yield of the classifier based on the number of patients who were indeed recommended to change or stop ≥1 prescription when reviewed. We compared this number with the number expected, if the ward round had reviewed a random 10%, 20%, or 30% patients receiving antibiotics.

### Ethical Considerations and Consent

This study received a favorable opinion from the West Midlands–Edgbaston Research Ethics Committee (reference 20/WM/0269). The requirement for informed consent was waived by the Research Ethics Committee as the study used pseudonymized data generated as part of routine care (reviewing prescriptions being part of day-to-day antibiotic stewardship).

## RESULTS

### Data Collection

The training set consisted of 2625 recommendations made by 40 infection specialists between September 2019 and April 2022. Recommendations related to 2414 prescriptions in 1487 patients. Of these recommendations, half (51%) were to continue the prescription as planned (n = 1326), 30% were to change it (n = 798), and 19% were to stop it (n = 501). The top antibiotic classes were β-lactams/penicillins (Anatomical Therapeutic Chemical classification [ATC] J01C; 33%), third-generation cephalosporins (ATC J01DD; 10%), and glycopeptides (J01XA; 9.7%). Neurology and neurosurgery specialties dominate the training dataset. Further details are available in Supplementary Material 3, while key features of the dataset are presented in Table 1.

**Table 1.** Descriptive Characteristics of the Training Dataset by Review Decision

Characteristic	Recommendations, No. (%)^a^			
	No Change (n = 1326)	Change (n = 798)	Stop (n = 501)	Total (n = 2625)
Unique patients, no	881	592	399	1487
Unique therapy episodes, no.	950	632	416	1686
Unique prescriptions, no.	1227	785	498	2414
Sex				
Female	575 (43)	354 (44)	246 (49)	1175 (45)
Male	751 (57)	444 (56)	255 (51)	1450 (55)
Age group				
18–44 y	266 (20)	153 (19)	95 (19)	514 (20)
45–59 y	347 (26)	158 (20)	120 (24)	625 (24)
60–74 y	422 (32)	274 (34)	148 (30)	844 (32)
≥75 y	291 (22)	213 (27)	138 (28)	642 (24)
Route of administration				
Oral	455 (34)	150 (19)	139 (28)	744 (28)
Parenteral	871 (66)	648 (81)	362 (72)	1881 (72)
Type of therapy (at time of review)				
Monotherapy	752 (57)	456 (57)	259 (52)	1467 (56)
Combination (2)	396 (30)	259 (32)	156 (31)	811 (31)
Combination (≥3)	178 (13)	83 (10)	86 (17)	347 (13)
Prescription indication (type of infection)				
Bloodstream/sepsis	202 (15)	114 (14)	57 (11)	373 (14)
Pneumonia	249 (19)	160 (20)	151 (30)	560 (21)
Other respiratory	7 (0.5)	9 (1.1)	6 (1.2)	22 (0.8)
Lower UTI	115 (8.7)	51 (6.4)	48 (9.6)	214 (8.2)
Upper UTI	54 (4.1)	38 (4.8)	8 (1.6)	100 (3.8)
Other GU	59 (4.4)	28 (3.5)	16 (3.2)	103 (3.9)
Endocarditis	26 (2.0)	9 (1.1)	2 (0.4)	37 (1.4)
CNS	133 (10)	75 (9.4)	36 (7.2)	244 (9.3)
GI/abdominal	132 (10.0)	93 (12)	60 (12)	285 (11)
Skin/soft tissue	64 (4.8)	54 (6.8)	24 (4.8)	142 (5.4)
Obstetrics	5 (0.4)	2 (0.3)	0 (0)	7 (0.3)
Bone/joint	47 (3.5)	13 (1.6)	7 (1.4)	67 (2.6)
Device/surgical site	49 (3.7)	29 (3.6)	22 (4.4)	100 (3.8)
Prophylaxis	16 (1.2)	9 (1.1)	14 (2.8)	39 (1.5)
Other/unknown	168 (13)	114 (14)	50 (10.0)	332 (13)
Prescription having a stop date (at time of review)	925 (70)	551 (69)	337 (67)	1813 (69)
Duration, median (IQR), d				
Prescription time elapsed at time of review	1.6 (0.7–3.1)	1.7 (0.7–2.9)	2.0 (0.7–4.5)	1.7 (0.7–3.4)
Prescription total duration	4.0 (2.5–7.5)	2.9 (1.4–5.4)	2.8 (1.1–4.9)	3.6 (1.9–6.6)
Therapy time elapsed at time of review	3.4 (1.7–8.4)	3.4 (1.7–7.0)	4.7 (1.9–8.4)	3.6 (1.7–7.9)
Total length of therapy	9.6 (5.5–20.1)	9.5 (5.4–17.8)	6.8 (3.9–12.1)	9.0 (5.0–17.1)

Abbreviations: CNS, central nervous system; GI, gastrointestinal; GU, genitourinary; IQR, interquartile range; UTI, urinary tract infection.

^a^Data represent no. (%) of recommendations unless otherwise specified in the characteristics column.

The prospective validation dataset was collected by 8 infection specialists during 97 PPR rounds between October 2021 and April 2022. On the day of each round, a software package ran automatically at 6 Am and extracted a systematic random sample of patients receiving systemic antibiotic therapy at the end of the preceding day. A total of 514 prescriptions belonging to 376 patients were extracted by the software package; 29 became ineligible due to the patient being discharged or therapy being stopped before the review took place. Of 475 prescriptions left to review, another 29 (6%) were missed. This means that a total of 446 PPR recommendations were recorded for 443 distinct prescriptions (including 3 that were reviewed on 2 separate PPRs) belonging to 338 patients. These could be aggregated to 358 patient-level reviews. Infection specialists recommended to continue 61% (n = 274), change 22% (n = 97), and stop 17% (n = 75) of them. The patients, prescriptions, and review recommendations in the prospective sample were very similar in those in the training and validation datasets (Supplementary Material 3).

### Model Tuning and Diagnostic Performance

**Figure 3.**
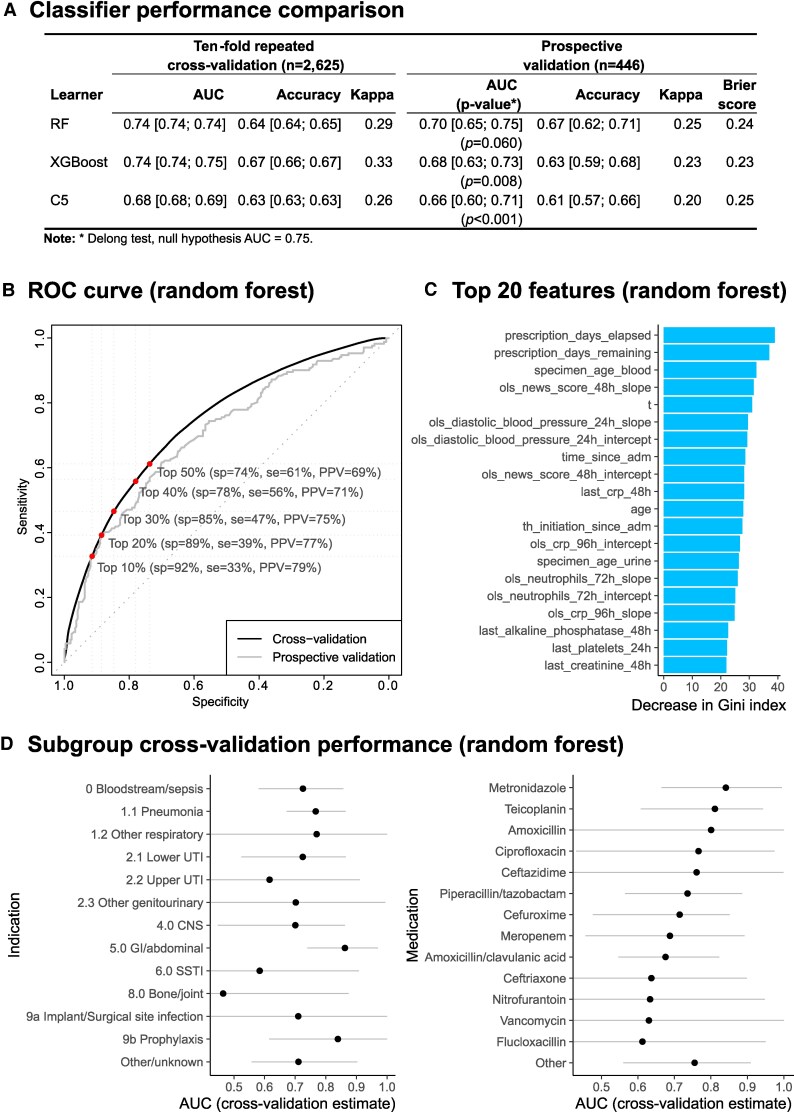
Cross-validation and prospective validation performance results. *A*, Binary classification performance (“stop” or “change” vs “no change”) estimates of the full area under the receiver operating characteristic (ROC) curve (AUC) for random forest (RF), C5, and XGBoost classifiers (with 95% confidence intervals [CIs]). *B*, ROC curve of the RF classifier. Abbreviations: PPV, positive predictive value; Se, sensitivity; Sp, specificity. *C*, Bar chart of the 20 features with highest-ranking importance. Abbreviations: CRP, C-reactive protein; NEWS, National Early Warning Score; OLS, ordinary least squares; T, time; TH, therapy. *D*, Subgroup AUC cross-validation estimates by prescription indication and drug. Abbreviations: CNS, central nervous system; SSTI, skin and soft-tissue infection; UTI, urinary tract infection.

Of 388 features considered (Supplementary Material 2), 131 were excluded due to near-zero variance, insufficient data completeness, or a correlation coefficient with other predictors exceeding 0.90. Three classifiers were fitted using the remaining 177 features: random forest, XGBoost, and C5. Optimal hyperparameters were selected based on AUCs using 10-fold cross-validation (see Supplementary Material 4). The binary performance (stop/change vs no change) of the final models is reported in Figure 3*A*. Cross-validation AUC was higher for the random forest and XGBoost classifiers, at 0.74. Validation in the prospective dataset found a better performance with the random forest classifier, with a slightly smaller drop in AUC at 0.70, compared with 0.68 for XGBoost. While calibration was sound under cross-validation, in the prospective validation dataset probabilities of stop/change decisions were negatively biased in all classifiers (with a Brier score of approximately 0.24), an indication of generalization bias in the models. Binary receiver operating characteristic curves for the random forest classifier are reproduced in Figure 3*B*, including sensitivity and specificity values at the top 5 deciles of the predicted probability of a stop/change recommendation. Under cross-validation, at a specificity of 75%, the model achieved a sensitivity of 60% and a PPV of 70%. Under prospective validation, for the same specificity, it achieved a sensitivity of 55% and a PPV of 58%.

Figure 3 *C* presents estimates of subgroup performance by clinical syndrome and drug class, with some suggestion that performance is highest in gastrointestinal/metronidazole indications but comparatively weaker in bone, soft-tissue, and upper urinary tract infections. Drugs with the lowest performance included flucloxacillin (first-line treatment for diabetic foot along with other soft-tissue infections), nitrofurantoin (first-line treatment for urinary infections), vancomycin (gastrointestinal indications). and ceftriaxone (central nervous system). Under cross-validation, performance was comparable across ethnic groups (Supplementary Material 8).

### Clinical Utility

The best classifier (random forest) was used to compute binary predictions (stop/change vs no change) for every prescription in the validation dataset. Those predictions were aggregated at the patient-review level (n = 358) by selecting the highest probability of stop/change recommendations among all prescriptions for a given patient. Actual recommendations recorded by infection specialists were also aggregated. A patient was reclassified as “no change” if all prescriptions currently active had that recommendation, as “discontinue therapy” if all prescriptions currently active had that recommendation, or “change” in all other cases.

According to this presentation, 41% (95% confidence interval, 36%–46%) of patients reviewed (145 of 358) were recommended to change or stop therapy. Table 2 tabulates these aggregated labels in the patients ranking highest on the classifier predictions. This simulates how the classifier could be used to prioritize PPR.

**Table 2.** Proportion of Patients With Recommendations to Stop or Change ≥1 Prescription Among the Top 3 Deciles of the Algorithm Prediction (Prospective Validation Dataset; 358 Patient-Level Review Decisions) and Expected Yield With or Without the Random Forest Classifier

Patient Category	Patients by Infection Specialist Recommendation, No. (%)^b^			Total No.	Patients by Expected Yield, No. (%)^b^	
	Discontinue Therapy	Change ≥1 Prescription	Continue, No Changes		Classifier-Driven Review	Review of Random Set^c^
Top 10%^a^	12 (34)	11 (31)	12 (34)	35	23 (66)	14 (41)
Top 20%^a^	24 (34)	25 (35)	22 (31)	71	49 (69)	29 (41)
Top 30%^a^	30 (28)	38 (36)	39 (36)	107	68 (64)	43 (41)
All patients	58 (16)	87 (24)	213 (59)	358	…	…

^a^Patients ranked by the maximum predicted probability of stop/change decisions using the random forest classifier.

^b^Row percentages.

^c^No. computed as total No × (58 + 87)/358.

For instance, in the 20% of patients with the highest predicted probability of stop/change recommendations (n = 71), infection specialists recommended changing or discontinuing therapy in 49 of 71 patients reviewed. This compares with an expected 29 had they reviewed 71 patients selected at random. In other words, if infection specialist could review only 20% of patients receiving antibiotics, the classifier would enable them to change treatment in 49 patients, 69% more than the 29 they would reach if approaching patients randomly.

### Feature Importance

The variables with the highest predictive importance for the random forest classifier are presented in Figure 3*B*, which largely overlapped the most important features in other classifiers (Supplementary Material 6): time since start of prescription, time remaining on prescription, time since start of therapy, NEWS2 score, blood pressure, time since a microorganism was last identified in blood, neutrophil counts, and C-reactive protein levels.

## DISCUSSION

In the median hospital, a third of patients receive antibiotics [4]. In our study, infection specialists deemed that 41% of patients receiving antibiotics needed to change or stop this therapy, similar to the prevalence cited in previous literature [5–7]. This means that approximately 1 in 10 inpatients is expected to benefit from a PPR consultation with an infection specialist. Infection specialists constitute a highly skilled but short-numbered workforce. We investigated the feasibility of using supervised machine learning to predict whether PPR would lead to a recommendation to stop or change a patient's antibacterial therapy using real-time electronic health records. The best classifier trained in a sample of 2625 prescription reviews had a moderate AUC of 0.70 in a prospective sample of 446 patients reviewed by infection specialists blinded to the prediction. Our evaluation suggested that such an algorithm to prioritize patients for review could increase the number of regimens optimized by specialists by 50%–66%.

This study focused on the task of ranking patients for PPR. Past research has been dominated by the complex engineering of rule-based expert systems to generate alerts [40–44]. The comprehensive diagnostic performance of those alerts was often not evaluated or published. One evaluation of a rule-based system tested in Sherbrooke, Quebec, found an accuracy of 79% in alerts issued for piperacillin-tazobactam prescriptions, measured against the decision made by a pharmacist reviewing the alert [17].

Three earlier studies have investigated the use of supervised machine learning. Bystritsky et al [45] used a large (n = 18 725) but imbalanced training dataset, and only 4% of therapy regimens corresponded to stop/change decisions. This was due to the same label being assigned to both patients who were reviewed and deemed to be on appropriate therapy and on patients who were not reviewed. Learning was performed at the level of patients, rather than prescriptions. These differences aside, findings from both studies concur: Bystritsky et al measured an AUC of 0.73–0.75 in a hold-out dataset, compared with 0.70 in our prospective validation dataset. This is a moderate performance level; for context, an AUC of 0.5 indicates no predictive performance, whereas an AUC of 1.0 indicates perfect predictive performance.

Goodman et al [46] also used a larger training dataset (n = 17 503) from an antimicrobial stewardship program, reviewing every prescription not discontinued after 3 days, with 24% of reviews leading to a change/discontinuation recommendation. Unlike in our study, prediction was at the level of patients, and features included ward-level characteristics (specialty, location, and presence of a ward pharmacist). Logistic regression and random forest classifiers, respectively, achieved AUCs of 0.68–0.72 and 0.75–0.77 in hold-out validation.

Tran-The et al [47] demonstrated similar performance in a more focused learning task. The authors assembled a training set (n = 27 677) by creating labels from records of discontinuation and de-escalation events exclusively in records of patients receiving ≥1 of 11 high-priority antibiotics (carbapenems, quinolones, vancomycin, cefepime, and piperacillin-tazobactam) subject to prior authorization by an infection specialist for durations exceeding 3 days. Point estimates of AUC were 0.80 for discontinuation and ranged from 0.69 to 0.81 for de-escalation labels. The potential use of such an algorithm is different from the approach in the previous 2 studies, but it demonstrates opportunities to create training datasets without manual labeling. Such data may have important uses for pretraining models destined for other applications.

Our study did not identify a clear dominant subset among all features examined. This means that the learning task requires extensive data modeling, a significant obstacle given the existing challenges in integrating healthcare data that have already been identified [48, 49].

The main strength of this study resides in the collection of a statistically representative and adequately powered validation dataset. Unlike the training set, the validation set was representative of wards and specialties where the classifier is susceptible to be used. Importantly, of 8 infection specialists involved, only 2 participated in the collection of the training dataset, which added some interrater variability to our evaluation and provided a very rigorous test of the classifier's generalizability. A majority of the validation dataset was collected after the training dataset and may be able to capture potential temporal drift in the classification. In particular, the training dataset was affected by 2 waves of the coronavirus disease 2019 pandemic (March–April 2020 and November 2020 to March 2021), during which pneumonia and bloodstream infections represented more than half of episodes of therapy, compared with a third at normal times.

The current study, however, was limited by the amount of training data available (n = 2625) relative to the complexity of the learning task: antibiotic therapy optimization is highly dependent on the infection and the class of drugs used. Many classes of antibiotics had <100 observations available. Both training and validation datasets were collected in a single site, which also limits our ability to evaluate the generalizability of the algorithms to other hospitals using different systems and observing different prescribing practices.

In conclusion, clinical systems have the potential to help a finite clinical workforce identify patients needing therapy optimization. Supervised machine learning, like rule-based expert systems, may play a role, but it is likely to require a large training dataset and extensive validation of performance and generalizability. Future research could attempt to develop models identifying/prioritizing patients according to the expected benefit from a review beyond just the predicted probability that their therapy regimen could be changed, by considering the risk of adverse events, such as drug reactions [50], or the promotion of drug resistance [51]. This holds the potential to support a wider workforce in optimizing antibiotic therapy—for example, ward pharmacists [13, 52].

## Supplementary Material

ofaf721_Supplementary_Data

## Supplementary Data

Supplementary materials are available at *Open Forum Infectious Diseases* online. Consisting of data provided by the authors to benefit the reader, the posted materials are not copyedited and are the sole responsibility of the authors, so questions or comments should be addressed to the corresponding author.

## Notes

***Acknowledgments.*** We thank Radha Asher, Lucy Bell, Malin Bergstrom, Anna Checkley, Arthur Clegg, Charles Coughlan, Tim Cutfield, Hanna Daire, Abhi Das, Sophia De Saram, Benedict Girling, Stephanie Harris, Vicky Johnston, David Kate, Ben Killingley, Sarah Logan, Diana Mabayoje, Irfaan Maan, Sarah Mahmoud, Emily Martyn, Michael Marks, Bruce McRae, Abdulrahman Mohamed, Steve Morris-Jones, Blanche Oguti, Preet Panesar, Trupti Patel, Naomi Platt, Gabriele Pollara, Alasdair Sinclair, Ana Soares, Tadhg Sullivan, Nel Swanepoel, Penelope Teoh, Clare Thakker, Chris Whitty, Constance Wraith, and many others who contributed to the SamurAI study.

***Author contributions*.** Conceptualization: P. F. D. M. and L. S. Methodology: P. F. D. M. Investigation: P. F. D. M., M. B., S. H., C. C., R. D., S. D., N. S., and L. S. Resources: M. B., S. H., C. C., R. D., S. D., and N. S. Software: P. F. D. M., H. C., and A. C. Formal analysis: P. F. D. M. Supervision: J. C. Writing: All authors.

***Disclaimer.*** The views expressed in this publication are those of the authors and not necessarily those of the National Institute for Health Research, NHSX, or the Department of Health and Social Care.

***Data availability*.** Applications for data access can be submitted to the University College London Hospitals NHS Foundation Trust Clinical and Research Informatics Unit (uclh.criu@nhs.net).

***Financial support***. This work was supported by the National Institute for Health Research (Artificial Intelligence, Stewardship of Antimicrobials using Real-Time Artificial Intelligence [SamurAI—phase 1; AI_AWARD01828] and research professorship NIHR302435 to L. S.); NHSX; the National Institute for Health and Care Research University College London Hospitals Biomedical Research Centre (including support to S. H. and L. S.).

***Potential conflicts of interest.*** All authors: No reported conflicts.
