# Supplementary material for: Supervised Machine Learning to Identify Hospital Inpatients Needing a Change of Antibiotic Therapy in Real Time: Preclinical Diagnostic Evaluation and Feasibility Study
Source: Open Forum Infect Dis. 2025 Nov 25;13(1):ofaf721. doi: 10.1093/ofid/ofaf721 (PMC12817996; doi:10.1093/ofid/ofaf721)
Supplement: ofaf721_Supplementary_Data [file ofaf721_supplementary_data.docx]

**Supervised machine learning to identify hospital inpatients needing a change of antibiotic therapy in real-time:**
**Preclinical diagnostic evaluation and feasibility study**

# Supplementary Materials

# Prescription indications

The UCLH electronic prescribing system mandates the recording of a therapeutic or prophylactic indication for all antibacterial orders. This involves two steps:

(1) the user first selects one of 15 high-level categories (broadly aligned to body system categories)

(2) the user then selects one indication (eg: community-acquired pneumonia) from a narrowed down set. An ‘other’ option allows the user to enter free text.

Table S1.1 presents indications and SNOMED CT concept codes used to represent both high-level categories, and the selected indication. Fifteen percent of prescriptions had free-text indications recorded, which were mapped to a SNOMED CT infection concept using fuzzy matching using a Snowstorm terminology server.

All SNOMED CT concepts were characterised into 82 binary features depending on whether they belong to wider definitions (for instance: lower respiratory infection) built using the SNOMED CT Expression Constraint Language (Table S2.1). Definitions were implemented using Snowstorm.

## Antibacterial prescription indication menu at UCLH with corresponding SNOMED CT concepts

| **Category** | **Indication** | **SNOMED CT concept** |
| --- | --- | --- |
| High-level category | Abdominal/GI | 715852004 |
| High-level category | Antimicrobial prophylaxis | 360271000 |
| High-level category | Bone/Joint | 312154004 |
| High-level category | Cardiovascular System | 128402005 |
| High-level category | Central Nervous System | 128117002 |
| High-level category | Ear/nose/throat | 54150009 |
| High-level category | Genitourinary | 189176002 |
| High-level category | Sepsis | 91302008 |
| High-level category | Skin/soft tissue | 95880003 |
| High-level category | Surgical site infection | 433202001 |
| Central Nervous System | Brain abscess | 441806004 |
| Central Nervous System | CNS implant infection | 473072000 |
| Central Nervous System | Encephalitis | 312215006 |
| Central Nervous System | Meningitis | 312216007 |
| Central Nervous System | Sub-galeal infection | 200727009 |
| Central Nervous System | Ventriculitis | 312217003 |
| Abdominal/GI | Appendicitis | 74400008 |
| Abdominal/GI | Bacterial gastroenteritis | 274080003 |
| Abdominal/GI | C. difficile (mild-moderate) | 186431008 |
| Abdominal/GI | C. difficile (severe) | 186431008 |
| Abdominal/GI | Cholecystitis/cholangitis | 82403002 |
| Abdominal/GI | Colitis (non-C. difficile) | 39341005 |
| Abdominal/GI | Diverticulitis | 271366000 |
| Abdominal/GI | H. pylori treatment | 281897000 |
| Abdominal/GI | Intra-abdominal collection | 235993005 |
| Abdominal/GI | Intra-abdominal sepsis (non-operative) | 128070006 |
| Abdominal/GI | Intra-abdominal sepsis (post-operative) | 308887002 |
| Abdominal/GI | Liver abscess | 27916005 |
| Abdominal/GI | Lower GI tract perforation | 56905009 |
| Abdominal/GI | Oesoophageal perforation | 23387001 |
| Abdominal/GI | Pancreatitis | 39205007 |
| Abdominal/GI | Small intestinal bacterial overgrowth | 446081009 |
| Abdominal/GI | Spontaneous bacterial peritonitis | 11836002 |
| Abdominal/GI | Variceal bleeding | 24807004 |
| Lung/CVS | Bronchiectasis | 879963005 |
| Lung/CVS | COPD, infective exacerbation | 196001008 |
| Lung/CVS | Empyema | 312682007 |
| Lung/CVS | Pneumonia (aspiration, community-onset) | 422588002 |
| Lung/CVS | Pneumonia (aspiration, hospital-onset) | 422588002 |
| Lung/CVS | Pneumonia (community, mild, CURB-65 0-1) | 385093006 |
| Lung/CVS | Pneumonia (community, moderate, CURB-65 1-2) | 385093006 |
| Lung/CVS | Pneumonia (community, severe, CURB-65 = 3) | 385093006 |
| Lung/CVS | Pneumonia (hospital, complex) | 425464007 |
| Lung/CVS | Pneumonia (hospital, simple) | 425464007 |
| Lung/CVS | Ventilator-associated pneumonia | 429271009 |
| Ear/nose/throat | Deep space neck infection | 6284004 |
| Ear/nose/throat | Mastoiditis | 52404001 |
| Ear/nose/throat | Otitis externa | 3135009 |
| Ear/nose/throat | Otitis media | 65363002 |
| Ear/nose/throat | Peritonsillar abscess/quinsy | 15033003 |
| Ear/nose/throat | Rhinosinusitis | 897656009 |
| Ear/nose/throat | Supraglottitis | 709663002 |
| Ear/nose/throat | Tonsilitis/pharyngitis | 405737000 |
| Ear/nose/throat | Discitis | 302935008 |
| Spinal | Epidural abscess | 61974008 |
| Spinal | Spinal osteomyelitis | 203241002 |
| Cardiovascular System | Endocarditis | 233850007 |
| Genitourinary | Asymptomatic bacteriuria | 720406004 |
| Genitourinary | Catheter-associated UTI (incl Stent, Nephrostomy, Other) | 700372006 |
| Genitourinary | Epididymitis / Epididymo-orchitis | 312224002 |
| Genitourinary | LOWER urinary tract infection (Cystitis) | 4009004 |
| Genitourinary | Pelvic inflammatory disease | 198130006 |
| Genitourinary | Prostatitis | 9713002 |
| Genitourinary | Prosthetic infection (AUS, Penile, Testicular, Mesh, Other) | 473056008 |
| Genitourinary | UPPER urinary tract infection (Pyelonephritis) | 422747000 |
| Genitourinary | Urethritis | 31822004 |
| Obstetric | Chorioamnionitis/pyrexia in labour | 11612004 |
| Obstetric | Endometritis | 78623009 |
| Obstetric | GBS prophylaxis | 405634003 |
| Obstetric | Post-partum infection | 178280004 |
| Obstetric | PPROM | 312974005 |
| Obstetric | Prophylaxis for instrumental deliveries | 236991000 |
| Obstetric | Sepsis in obstetric infections | 91302008 |
| Surgical site infection | Deep incisional/wound infection | 213286009 |
| Surgical site infection | Organ or space surgical site infection | 433202001 |
| Surgical site infection | Superficial incisional/wound infection | 213287000 |
| Skin/soft tissue | Cellulitis | 402929007 |
| Skin/soft tissue | Cellulitis (facial/orbital) | 128277003 |
| Skin/soft tissue | Diabetic foot infection | 280137006 |
| Skin/soft tissue | Mastitis/breast abscess | 266579006 |
| Skin/soft tissue | Necrotising fasciitis | 52486002 |
| Bone/Joint | Orthopaedic implant infection | 473053000 |
| Bone/Joint | Osteomyelitis | 60168000 |
| Bone/Joint | Osteomyelitis (non-spinal) | 60168000 |
| Bone/Joint | Septic arthritis | 48245008 |
| Sepsis | Neutropenic sepsis | 443980004 |
| Sepsis | Sepsis (unknown source) | 91302008 |
| Sepsis | Sepsis in obstetric infections | 91302008 |
| Sepsis | Sepsis in sickle cell disease/thalassaemia | 91302008 |
| Miscellaneous | Atypical mycobacterial infection | 111812000 |
| Miscellaneous | Bacteraemia | 5758002 |
| Miscellaneous | Fever of unknown origin | 7520000 |
| Miscellaneous | Line-infection | 736152001 |
| Miscellaneous | Malaria | 61462000 |
| Miscellaneous | MRSA suppression | 266096002 |
| Miscellaneous | PCP treatment | 415125002 |
| Miscellaneous | Tuberculosis/TB | 56717001 |
| Antimicrobial prophylaxis | PCP prophylaxis | 415125002 |
| Antimicrobial prophylaxis | PCP/toxoplasmosis prophylaxis | 415125002 |
| Antimicrobial prophylaxis | Pneumococcal prophylaxis | 16814004 |
| Antimicrobial prophylaxis | Splenectomy | 234319005 |
| Antimicrobial prophylaxis | Surgical prophylaxis | 387713003 |
| Other | <free text> | Mapped using Snowstorm |

## SNOMED CT Expression Constraint Language (ECL) definition of prescription indication concepts

| **Feature** | **SNOMED CT ECL definition** |
| --- | --- |
| soft_tissues | * : 363698007 = <<87784001 \| Soft tissues (body structure) \| |
| digestive | * : 363698007 = <<86762007 \| Structure of digestive system (body structure) \| |
| hand | * : 363698007 = <<85562004 \| Hand structure (body structure) \| |
| lower_respiratory | * : 363698007 = <<82094008 \| Lower respiratory tract structure (body structure) \| |
| abdomen | * : 363698007 = <<818983003 \| Structure of abdominopelvic cavity and/or content of abdominopelvic cavity and/or anterior abdominal wall (body structure) \| |
| mediastinal | * : 363698007 = <<72410000 \| Mediastinal structure (body structure) \| |
| genital | * : 363698007 = <<71934003 \| Genital structure (body structure) \| |
| orbital | * : 363698007 = <<714483002 \| Structure of orbital region (body structure) \| |
| eye | * : 363698007 = <<371398005 \| Eye region structure (body structure) \| |
| head | * : 363698007 = <<69536005 \| Head structure (body structure) \| |
| upper_respiratory | * : 363698007 = <<58675001 \| Upper respiratory tract structure (body structure) \| |
| foot | * : 363698007 = <<56459004 \| Foot structure (body structure) \| |
| nasopharynx | * : 363698007 = <<312535008 \| Pharynx and/or larynx structures (body structure) \| |
| oropharynx | * : 363698007 = <<312533001 \| Mouth and/or pharynx structures (body structure) \| |
| upper_urinary | * : 363698007 = <<304582006 \| Structure of upper urinary system (body structure) \| |
| inner_ear | * : 363698007 = <<302463004 \| Inner ear and/or Eustachian canal structures (body structure) \| |
| musculoskeletal | * : 363698007 = <<26107004 \| Structure of musculoskeletal system (body structure) \| |
| middle_ear | * : 363698007 = <<25342003 \| Middle ear structure (body structure) \| |
| genitourinary | * : 363698007 = <<21514008 \| Structure of genitourinary system (body structure) \| |
| central_nervous | * : 363698007 = <<21483005 \| Structure of central nervous system (body structure) \| |
| respiratory | * : 363698007 = <<20139000 \| Structure of respiratory system (body structure) \| |
| lower_urinary | * : 363698007 = <<19787009 \| Lower urinary tract structure (body structure) \| |
| urinary | * : 363698007 = <<122489005 \| Urinary system structure (body structure) \| |
| immune | * : 363698007 = <<116003000 \| Structure of immune system (body structure) \| |
| cardiovascular | * : 363698007 = <<113257007 \| Structure of cardiovascular system (body structure) \| |
| external_auditory_canal | * : 363698007 = <<84301002 \| External auditory canal structure (body structure) \| |
| oral_hard_tissue | * : 363698007 = <<272660004 \| Oral hard tissue structure (body structure) \| |
| breast | * : 363698007 = <<76752008 \| Breast structure (body structure) \| |
| large_intestine | * : 363698007 = <<14742008 \| Structure of large intestine (body structure) \| |
| pleural_sac | * : 363698007 = <<116006008 \| Pleural sac structure (body structure) \| |
| nail_unit | * : 363698007 = <<770802007 \| Nail unit structure (body structure) \| |
| dysreg_host_response | <404684003 \| Clinical finding (finding) \| :   { 370135005 \|Pathological process (attribute)\| = 769256002 \|Dysregulated host response (qualifier value)\| } |
| associated_sepsis | (<<110276005 \| Deep mycosis (disorder) \| OR   <<40733004 \| Infectious disease (disorder) \| OR   <<473130003 \| Suspected infectious disease (situation) \| OR  <<128045006 \| Cellulitis (disorder) \|) :  (42752001 \| Due to \| = << 91302008 \|Sepsis\|) OR (47429007 \| Associated with \| = << 91302008 \|Sepsis\|) |
| infection_covid | <<186747009 \| Coronavirus infection (disorder) \| |
| infection_bloodstream | << 434156008\|Infectious agent in bloodstream\| |
| associated_device | (<<110276005 \| Deep mycosis (disorder) \| OR   <<40733004 \| Infectious disease (disorder) \| OR   <<473130003 \| Suspected infectious disease (situation) \| OR  <<128045006 \| Cellulitis (disorder) \|) :   47429007 \| Associated with (attribute) \| = << 49062001 \| Device (physical object) \| |
| morphology_carbuncle | (<<110276005 \| Deep mycosis (disorder) \| OR   <<40733004 \| Infectious disease (disorder) \| OR   <<473130003 \| Suspected infectious disease (situation) \| OR  <<128045006 \| Cellulitis (disorder) \|) :   116676008 \| Associated morphology (attribute) \| = 41570003 \| Carbuncle (morphologic abnormality) \| |
| cause_bacterial | (<<110276005 \| Deep mycosis (disorder) \| OR   <<40733004 \| Infectious disease (disorder) \| OR   <<473130003 \| Suspected infectious disease (situation) \| OR  <<128045006 \| Cellulitis (disorder) \|) :   246075003 \| Causative agent (attribute) \| = << 409822003 \| Domain Bacteria (organism) \| |
| cause_fungal | (<<110276005 \| Deep mycosis (disorder) \| OR   <<40733004 \| Infectious disease (disorder) \| OR   <<473130003 \| Suspected infectious disease (situation) \| OR  <<128045006 \| Cellulitis (disorder) \|) :   246075003 \| Causative agent (attribute) \| = << 414561005 \| Kingdom Fungi (organism) \| |
| cause_viral | (<<110276005 \| Deep mycosis (disorder) \| OR   <<40733004 \| Infectious disease (disorder) \| OR   <<473130003 \| Suspected infectious disease (situation) \| OR  <<128045006 \| Cellulitis (disorder) \|) :   246075003 \| Causative agent (attribute) \| = << 49872002 \| Virus (organism) \| |
| cause_parasitic | (<<110276005 \| Deep mycosis (disorder) \| OR   <<40733004 \| Infectious disease (disorder) \| OR   <<473130003 \| Suspected infectious disease (situation) \| OR  <<128045006 \| Cellulitis (disorder) \|) :  370135005 \| Pathological process (attribute) \| = << 442614005 \| Parasitic process (qualifier value) \| |
| pneumonia | <<233604007 \| Pneumonia (disorder) \| |
| resp_tuberculosis | <<700272008 \| Tuberculosis of respiratory system (disorder) \| |
| renal_failure | <<42399005 \| Renal failure syndrome (disorder) \| |
| neonatal | (<<110276005 \| Deep mycosis (disorder) \| OR   <<40733004 \| Infectious disease (disorder) \| OR   <<473130003 \| Suspected infectious disease (situation) \| OR  <<128045006 \| Cellulitis (disorder) \|) :  246454002 \| Occurrence (attribute)\| = 255407002 \|Neonatal (qualifier value)\| |
| obstetrics | <<248982007 \| Pregnancy, childbirth and puerperium finding (finding) \| |
| suspected | (<<110276005 \| Deep mycosis (disorder) \| OR   <<40733004 \| Infectious disease (disorder) \| OR   <<473130003 \| Suspected infectious disease (situation) \| OR  <<128045006 \| Cellulitis (disorder) \|) :  408729009 \|Finding context (attribute)\| = 415684004 \|Suspected (qualifier value)\| |
| gas_gangrene | <<80466000 \| Gas gangrene (disorder) \| |
| infection_systemic_fung | <<110276005 \| Deep mycosis (disorder) \| OR <<399314004 \| Systemic mycosis (disorder) \| |
| finding_any | (<<110276005 \| Deep mycosis (disorder) \| OR   <<40733004 \| Infectious disease (disorder) \| OR   <<473130003 \| Suspected infectious disease (situation) \| OR  <<128045006 \| Cellulitis (disorder) \|) :   363698007 \|Finding site (attribute)\| = <113343008 \| Body organ structure (body structure) \| |
| influenza | <<6142004 \| Influenza (disorder) \| |
| cns_abscess | <<735554004 \| Infection causing abscess of central nervous system (disorder) \| |
| meningitis | <<7180009 \| Meningitis (disorder) \| |
| encephalitis | <<45170000 \| Encephalitis (disorder) \| |
| laryngitis | <<45913009 \|Laryngitis (disorder)\| |
| pharyngitis | <<405737000 \| Pharyngitis (disorder) \| |
| otitis_media | <<65363002 \| Otitis media (disorder) \| |
| sinusitis | <<36971009 \| Sinusitis (disorder) \| |
| endocarditis | <<56819008 \| Endocarditis (disorder) \| |
| myocarditis | <<50920009 \| Myocarditis (disorder) \| |
| mastoiditis | <<52404001 \| Mastoiditis (disorder) \| |
| necrotizing_fasciitis | <<52486002 \| Necrotizing fasciitis (disorder) \| |
| cellulitis | <<128045006 \| Cellulitis (disorder) \| |
| mastitis | <<23623005 \| Infective mastitis (disorder) \| |
| ssi | <<12246311000119109 \| Infection following procedure (disorder) \| |
| pancreatitis | <<75694006 \| Pancreatitis (disorder) \| |
| enteritis | <<64613007 \| Inflammation of small intestine (disorder) \| |
| gastritis | <<4556007 \| Gastritis (disorder) \| |
| hepatitis | <<128241005 \| Inflammatory disease of liver (disorder) \| |
| colitis | <<64226004 \| Colitis (disorder) \| |
| appendicitis | <<74400008 \| Appendicitis (disorder) \| |
| cholangitis | <<82403002 \| Cholangitis (disorder) \| |
| peritonitis | <<48661000 \| Peritonitis (disorder) \| |
| large | <<737345002 \| Infection of large intestine (disorder) \| |
| cholecystitis | <<76581006 \| Cholecystitis (disorder) \| |
| hiv | <<86406008 \| Human immunodeficiency virus infection (disorder) \| |
| measles | <<14189004 \| Measles (disorder) \| |
| rubella | <<36653000 \| Rubella (disorder) \| |
| mumps | <<36989005 \| Mumps (disorder) \| |
| varicella | <<38907003 \| Varicella (disorder) \| |
| erythema_infection | <<34730008 \| Primate erythroparvovirus 1 infection (disorder) \| |
| cdi | <<186431008 \| Clostridioides difficile infection (disorder) \| |

# Feature definition and selection

## Definition and number of training features

| Name | Total number (N) | Number selected  (N) | Definition and selection | % missing (training dataset) |
| --- | --- | --- | --- | --- |
|  |  |  | **Therapy characteristics** |  |
| Hours elapsed since start of therapy | 1 | 1 | - | 0 |
| Hours elapsed since start of prescription | 1 | 1 | - | 0 |
| Hours remaining on prescription | 1 | 1 | Missing = no stop date. Hardcoded as -99 in random forest classifier. | 31 |
| Hours elapsed since admission | 1 | 1 | - | 3 |
| Hours elapsed between admission and beginning of therapy | 1 | 1 | Can correlate with hospital-acquired infections. | 3 |
| Prescription number | 1 | 1 | Ranking prescription by authoring time since initiation of therapy (first, second, third, etc.). | 0 |
| Prescription AWaRe class | 1 | 1 | Access; Watch; Reserve; Other. | 0 |
| Prescription route of administration | 1 | 1 | Binary (parenteral/oral). | 0 |
| Therapy doses in grams for all medication orders currently active | 16 | 16 | Grouped into 16 drug classes from the AMR package [10]: Beta-lactams/penicillins; Glycopeptides; Aminoglycosides; Quinolones; Carbapenems; Trimethoprims; Macrolides/lincosamides; Cephalosporins (1st gen.); Cephalosporins (2nd gen.); Cephalosporins (3rd gen.); Cephalosporins (4th gen.); Tetracyclines; Oxazolidinones; Antimycobacterials; Amphenicols; Polymyxins; Other antibacterials. | 0 |
| Prescription drug | 1 | 1 | AMR package [3] antibiotic code. Only the 13 most frequent values from the training set were kept: Amoxicillin/clavulanic acid, Amoxicillin, Ceftazidime, Ciprofloxacin, Ceftriaxone, Cefuroxime, Flucloxacillin, Meropenem, Metronidazole, Nitrofurantoin, Teicoplanin, Piperacillin/tazobactam, Vancomycin. All other values coded as ‘Other’. | 0 |
| Prescription drug class | 16 | 16 | Grouped into 16 drug classes from the AMR package [10] (see above). | 0 |
| Prescription frequency | 1 | 1 | Daily frequency with one-off hardcoded as -1 and *pro re nata* as -9. | 0 |
| Prescription indication | 82 | 24 | Binary variables. See Table 2.  2 dropped due to excessive correlation, 58 dropped due to near-zero variance. | 0 |
|  |  |  | **Patient characteristics** |  |
| Age on admission | 1 | 1 | - | 0 |
| Sex | 1 | 1 | - | 0 |
| Ethnic group | 6 | 6 | 5 categories. Missing coded as ‘unknown’. | 21 |
| Charlson morbidity index weight of any past recorded diagnosis of a chronic morbidity | 14 | 5 | 0 = not recorded; 1-6 = maximum comorbidity weight [11]. Grouped by comorbidity: AIDS/HIV, liver disease, peripheral vascular disease; cerebrovascular disease; cancer; diabetes; renal disease; dementia; hemiplegia or paraplegia; congestive heart failure; acute myocardial infarction; pulmonary disease, peptic ulcer disease, rheumatoid disease. | 0 |
| Infection diagnoses | 83 | 21 | Encounter diagnoses so far in the admission, recategorized in the same way as prescription indications.  2 dropped due to excessive correlation, 60 dropped due to near-zero variance | 0 |
|  |  |  | **Microbiology** |  |
| Days since latest specimen sent for culture | 4 | 4 | By specimen type (blood; urine; swab; other fluid/tissue). Only specimens sampled in the last 30 days are included. Absence of specimen hardcoded as –‘999. | 27-47 |
| Days since latest pathogen isolated | 4 | 4 | By specimen type (blood; urine; swab; other fluid/tissue). Only specimens sampled in the last 30 days are included. Absence of isolate hardcoded as -999. | 76-87 |
| Any organisms isolated known to exhibit common resistance to all medication currently prescribed? | 1 | 1 | As per EUCAST intrinsic resistance phenotypes [2] imputed using the AMR package [3]. Binary variable (0/1) by specimen type (blood; urine; swab; other fluid/tissue). Absence of isolate/specimen hardcoded as -999. | 76-87 |
| Any organisms isolated found to be resistant to all medication currently prescribed with susceptibility testing? | 5 | 5 | Same as above, but antibiotic susceptibility testing are incorporated (results usually available 24-48 hours later). Binary variable (0/1) by specimen type (blood; urine; swab; other fluid/tissue). Inferred using EUCAST expert rules [2], using antibiotic susceptibility results if available. Only pathogens isolated in the last 30 days are included. Absence of isolate/specimen hardcoded as -999. | 79-90 |
| Any multidrug resistant organism found? | 5 | 5 | Binary variable (0/1) coding multidrug resistance as defined by Magiorakos et al. [12].  By specimen type (blood; urine; swab; other fluid/tissue). Only pathogens isolated in the last 30 days are included. | 79-90 |
| Urine leukocyte count: latest value in last 96 hours | 1 | 1 | Low boundary of qualitative value (<1; 1-15; 16-249; >250 10^6/L). | 53 |
| Urine yeast count: latest value in last 96 hours | 1 | 1 | Low boundary of qualitative value (<1; 1-15; 16-249; >250 10^6/L). | 53 |
|  |  |  | **Haematology** |  |
| Neutrophil count: slope, intercept, number of readings in last 72 hours | 3 | 3 | - | 24 |
| Neutrophil count: latest value in last 24 hours | 1 | 1 | - | 27 |
| Lymphocyte count: latest value in last 24 hours | 1 | 1 | - | 27 |
| Neutrophil:lymphocyte ratio: latest value in last 24 hours | 1 | 1 | - | 27 |
| Monocyte count: latest value in last 24 hours | 1 | 1 | - | 27 |
| Eosinophil count: latest value in last 24 hours | 1 | 1 | - | 28 |
| Basophil count: latest value in last 24 hours | 1 | 1 | - | 28 |
| Erythrocyte count: latest value in last 24 hours | 1 | 1 | - | 26 |
| Nucleated red blood cell count: latest value in last 24 hours | 1 | 1 | - | 27 |
| Nucleated red blood cell (%): latest value in last 24 hours | 1 | 1 | - | 27 |
| Erythrocyte distribution width: latest value in last 24 hours | 1 | 1 | - | 26 |
| Platelet count: latest value in last 24 hours | 1 | 1 | - | 26 |
| Platelet mean volume | 1 | 1 | - | 28 |
| Erythrocyte sedimentation rate: latest value in last 96 hours | 1 | 0 | Dropped due to near-zero variance. | 91 |
| Immature granulocyte count: latest value in last 96 hours | 1 | 0 | Dropped due to near-zero variance. | 86 |
| Immature granulocyte %: latest value in last 96 hours | 1 | 1 | - | 86 |
| Haemoglobin: latest value in last 24 hours | 1 | 1 | - | 26 |
| C-reactive protein: slope, intercept, number of readings in last 96 hours | 1 | 1 | - | 24 |
| C-reactive protein: latest value in last 48 hours | 1 | 1 | - | 17 |
| MCH: latest value in last 24 hours | 1 | 1 | - | 26 |
| MCHC: latest value in last 24 hours | 1 | 1 | - | 26 |
| MCT: latest value in last 24 hours | 1 | 1 | - | 26 |
| MCV: latest value in last 24 hours | 1 | 1 | - | 26 |
| HCT: latest value in last 24 hours | 1 | 1 | - | 26 |
| Urea: latest value in last 72 hours | 1 | 1 | - | 65 |
| Calcium: latest value in last 24 hours | 1 | 1 | - | 49 |
| Potassium: latest value in last 24 hours | 1 | 1 | - | 26 |
| Sodium: latest value in last 24 hours | 1 | 1 | - | 26 |
| Bilirubin: latest value in last 48 hours | 1 | 1 | - | 27 |
| Creatinine: latest value in last 48 hours | 1 | 1 | - | 11 |
| Estimated glomerular filtration rate: latest value in last 48 hours | 1 | 1 | Based on CKD-EPI Creatinine Equation. | 11 |
| Alkaline phosphatase: latest value in last 48 hours | 1 | 1 | - | 26 |
| Alanine aminotransferase: latest value in last 48 hours | 1 | 1 | - | 27 |
|  |  |  | **Physiology** |  |
| Temperature: latest value in last 8 hours | 1 | 1 | - | 6 |
| Temperature: counts of readings under/within/over [36.0; 38.0] ⁰C in last 24 hours | 3 | 3 | - | <1 |
| Respiration rate: counts of readings under/strictly over 20/min | 2 | 2 | - | <1 |
| Latest respiratory assistance status in 24 hours | 1 | 1 | On air room or oxygen | 2 |
| Pulse oximetry: number of values under/within/over [90%; 95%] range in last 12 hours | 3 | 3 | - | <1 |
| PaO2/FiO2 ratio: latest value in last 24 hours | 1 | 1 | - | 90 |
| Diastolic blood pressure: slope, intercept, number of readings over last 24 hours | 3 | 3 | - | 1 |
| Heart rate: number of readings strictly under/over 90/min in last 12 hours | 2 | 2 | - | <1 |
| Glasgow Coma Score: latest value in last 24 hours | 1 | 1 | - | 28 |
|  |  |  | **Risk scores** |  |
| NEWS 2 score: latest values in last 8 hours | 1 | 1 | - | 10 |
| NEWS 2 score: slope, intercept, number of readings in last 48 hours | 3 | 3 | - | 3 |
| SOFA score: latest value in last 24 hours | 1 | 1 | - | 77 |
| SOFA score: latest value in last 72 hours | 1 | 1 | - | 75 |

# Characteristics of the training and validation datasets

## Characteristics of post-prescription review decisions

|  | **Training dataset**  (N=2,625) | **Validation dataset**  (N=446) |
| --- | --- | --- |
| **Numbers** |  |  |
| Distinct patients (N) | 1,487 | 327 |
| Distinct therapy episodes (N) | 1,686 | 338 |
| Distinct prescriptions (N) | 2,414 | 443 |
| **Sex** |  |  |
| Female | 1,175 (45%) | 228 (51%) |
| Male | 1,450 (55%) | 218 (49%) |
| **Age** |  |  |
| 18-44 years | 514 (20%) | 100 (22%) |
| 45-59 years | 625 (24%) | 96 (22%) |
| 60-74 years | 844 (32%) | 118 (26%) |
| 75+ years | 642 (24%) | 132 (30%) |
| **Mode of administration** |  |  |
| Oral | 744 (28%) | 175 (39%) |
| Parenteral | 1,881 (72%) | 271 (61%) |
| **Type of therapy (at time of review)** |  |  |
| Monotherapy | 1,467 (56%) | 231 (52%) |
| In combination with 1 other prescription | 811 (31%) | 166 (37%) |
| In combination 2+ other prescriptions | 347 (13%) | 49 (11%) |
| **Prescription indication** |  |  |
| Bloodstream/sepsis | 373 (14%) | 35 (8%) |
| Pneumonia | 560 (21%) | 87 (20%) |
| Other respiratory | 22 (0.8%) | 12 (3%) |
| Lower UTI | 214 (8.2%) | 30 (7%) |
| Upper UTI | 100 (3.8%) | 16 (4%) |
| Other genitourinary | 103 (3.9%) | 22 (5%) |
| Endocarditis | 37 (1.4%) | 4 (1%) |
| CNS | 244 (9.3%) | 21 (5%) |
| GI/abdominal | 285 (11%) | 100 (22%) |
| Skin/soft tissue | 142 (5.4%) | 21 (5%) |
| Obstetrics | 7 (0.3%) | 8 (2%) |
| Bone/joint | 67 (2.6%) | 4 (1%) |
| Device/surgical site infection | 100 (3.8%) | 14 (3%) |
| Prophylaxis | 39 (1.5%) | 3 (1%) |
| Other/unknown | 332 (13%) | 22 (5%) |
| **Prescription has a stop date** (at time of review) | 1,813 (69%) | 345 (77%) |
| **Prescription time elapsed** (days at time of review)  median (IQR) | 1.7 (0.7, 3.4) | 1.9 (1.0; 3.6) |
| **Prescription total duration**  median (IQR) | 3.6 (1.9, 6.6) | 3.5 (2.2; 5.4) |
| **Therapy time elapsed** (days at time of review)  median (IQR) | 3.6 (1.7, 7.9) | 3.6 (1.8; 6.8) |
| **Total length of therapy (days)**  median (IQR) | 9.0 (5.0, 17.1) | 7.3 (4.3; 12.4) |
| **Review decision** |  |  |
| Continue | 1,326 (51%) | 274 (61%) |
| Change | 798 (30%) | 97 (22%) |
| Stop | 501 (19%) | 75 (17%) |
| **Drug group** |  |  |
| Aminoglycosides | 19 (0.7%) | 4 (0.9%) |
| Antimycobacterials | 24 (0.9%) | 7 (1.6%) |
| Betalactams/penicillins | 875 (33%) | 149 (33%) |
| Carbapenems | 194 (7.4%) | 19 (4.3%) |
| Cephalosporins 1st gen | 3 (0.1%) | 2 (0.4%) |
| Cephalosporins 2nd gen | 226 (8.6%) | 63 (14%) |
| Cephalosporins 3rd gen | 268 (10%) | 26 (5.8%) |
| Glycopeptides | 255 (9.7%) | 25 (5.6%) |
| Macrolides/lincosamides | 82 (3.1%) | 21 (4.7%) |
| Oxazolidinones | 43 (1.6%) | 3 (0.7%) |
| Quinolones | 165 (6.3%) | 32 (7.2%) |
| Tetracyclines | 56 (2.1%) | 11 (2.5%) |
| Trimethoprims | 60 (2.3%) | 13 (2.9%) |
| Other | 355 (14%) | 71 (16%) |

# Hyperparameters

Optimal hyperparameters were selected based on area under the curve using 10-fold cross-validation (5 iterations) conducted in the caret library using the search grids outlined in Table S4.1.

## Hyperparameter search grids

| **Classifier** | **Search grid** | **Best fitting hyperparamaters** |
| --- | --- | --- |
| Random forest | number of trees = 1000  mtry = [5, 15, 20, 30, 50, 100, 200, 400] | mtry = 20 |
| C5 | trials = [20, 35, 50, 75, 100]  winnow = [T, F] | trials = 100  winnow = F |
| xgboost | *Step 1*  nrounds = [200, 300]  max_depth = [1, 3, 5]  eta = [1.0, 0.1, 0.05]  gamma = [0, 0.5, 1, 2, 5]  min_child_weight = [1, 3, 5]  subsample = .8  colsample_bytree = .8 |  |
|  | *Step 2*  nrounds = 50 to 500, by increments of 50  max_depth = 50  eta = 0.05  gamma = 0  min_child_weight = 1  subsample = .8  colsample_bytree = .8 |  |
|  | *Step 3*  nrounds = 200  max_depth = [5, 10, 20, 30, 50]  eta = 0.05  gamma = 0  min_child_weight = 1  subsample = .8  colsample_bytree = .8 | nrounds = 200  max_depth = 50  eta = 0.05  gamma = 0  min_child_weight = 1  subsample = .8  colsample_bytree = .8 |

# Receiver operating characteristics


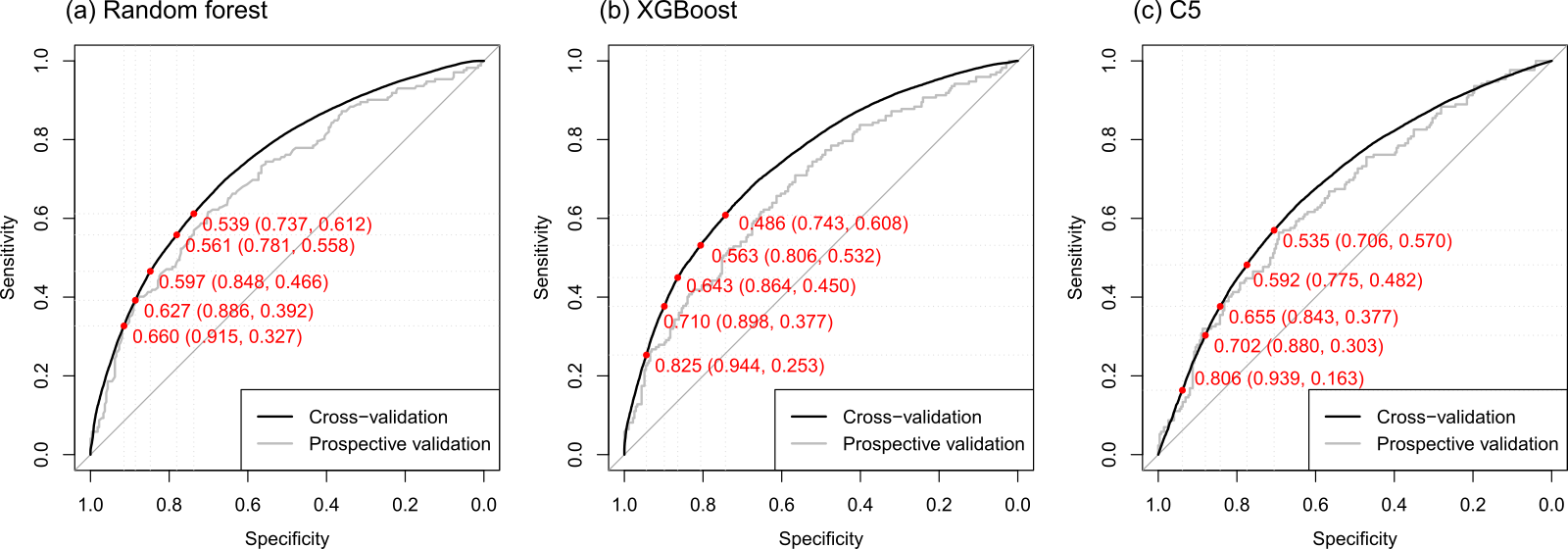


## Figure S5.1 Binary receiver operating characteristics curves of (a) random forest, (b) XGBoost and (c) C5 classifiers (‘stop’/’change’ vs ‘no change’) under cross-validation and prospective validation, reporting cut-offs (sensitivity, specificity) at cross-validation prediction deciles 5 to 9

Alt text: Plots depicting sensitivity against specificity for each classifier.

# Feature importance

## Top 20 features in decreasing order of importance by classifier

| **Rank** | **Random forest**  **Mean decrease in Gini** | **XGBoost**  **% gain from feature split** | **C5**  **% tree splits** |
| --- | --- | --- | --- |
| 1 | Hours elapsed since start of prescription | Hours remaining on prescription | Calendar year |
| 2 | Hours remaining on prescription | Hours elapsed since start of prescription | Hours elapsed since start of prescription |
| 3 | Days since latest blood specimen sent for culture | C-reactive protein: latest value | Hours elapsed since start of therapy |
| 4 | NEWS2 score: slope | Diastolic blood pressure: intercept | Indication: pneumonia |
| 5 | Hours elapsed since start of therapy | Diastolic blood pressure: slope | Days since latest blood specimen sent for culture |
| 6 | Diastolic blood pressure: slope | NEWS2 score: slope | Prescription number |
| 7 | Diastolic blood pressure: intercept | Days since latest blood specimen sent for culture | Ethnic group: Black |
| 8 | Hours elapsed since admission | NEWS2 score: intercept | Indication: bacterial infection |
| 9 | NEWS 2 score: intercept | Hours elapsed since admission | Indication: attributed to any body system |
| 10 | C-reactive protein: latest value | Neutrophil count: slope | Heart rate: number readings <90/min |
| 11 | Age | Hours elapsed since start of therapy | Hours remaining on prescription |
| 12 | Hours elapsed between admission and beginning of therapy | Hours elapsed between admission and beginning of therapy | Heart rate: number readings >90/min |
| 13 | C-reactive protein: intercept | C-reactive protein: slope | Charlson: cancer |
| 14 | Days since latest urine specimen sent for culture | Neutrophil count: intercept | Ethnic group: unknown |
| 15 | Neutrophil count: slope | C-reactive protein: intercept | Neutrophils: number of readings |
| 16 | Neutrophil count: intercept | Creatinine: latest value in last 48 hours | Drug: metronidazole |
| 17 | C-reactive protein: slope | Temperature: latest value | Diastolic blood pressure: number of readings |
| 18 | Alkaline phosphatase: latest value in last 48 hours | Alkaline phosphatase: latest value in last 48 hours | Age |
| 19 | Platelet count: latest value in last 24 hours | Alanine aminotransferase: latest value in last 48 hours | Charlson: cerebrovascular |
| 20 | Creatinine: latest value in last 48 hours | Age | Administration route |

# Target performance

**Objective**

This section reports on the a priori study design justifications of the performance requirements of the proposed device. A target performance level was specified as part of the funding application and subsequent study protocol, which underpinned the sample size calculation of the prospective validation data collection.

An AUC target was set as the lowest value that would still be compatible with doubling the yield of post-prescription reviews. **Yield was defined as the absolute number of antibiotics changed** when using the device. It is compared to what would happen without the device, if we assume an identical number of patients *k* are reviewed.

**Yield with the device** *Y_1_* = TP = P * sensitivity

where

- TP denotes the number of true positives,
- P the number of positives, that is, the number of patients receiving inappropriate antibiotics (TP + false negatives)
- sensitivity = TP / P, and is a function of the number of patients reviewed *k*.

**Yield without the device** *Y_0_* = 0.3 * *k*, where 0.3 is the prevalence of inappropriate prescriptions.

**Number of patients reviewed *k***

Our measure of yield revolved around how many patients are reviewed. Engagement with stakeholders and shadowing of antimicrobial stewardship ward rounds suggested that *k* would be strongly influenced by human factors. From a user perspective (microbiologist and pharmacists), a good use of time meant minimising the time reviewing antibiotics where no change was required.

Based on shadowing antimicrobial stewardship rounds, it was expected clinicians adopting the device would be very willing to review antibiotics when the proportion of true positive (PPV) was above 70%. From a workforce productivity perspective, this is a plausible investment of time for an intervention expected to shorten hospitalisation duration, given the amount of staff resourcing and beds that would be saved.

We derived the value of *k* (and the corresponding sensitivity) corresponding to reasonable use conditions from the fixed PPV value of 0.70, with the help of simulations.

PPV is a function of TP, the number of true positives, and FP the number of false negatives:

PPV = TP / (TP + FP) = 0.70 (1)

Since TP + FP is the number of patients to be reviewed, we substitute the notation *k*

TP / *k* = 0.70 (2)

Therefore

*k* = TP / 0.70 (3)

TP is the product of P, the number patients receiving inappropriate antibiotics, with the sensitivity at the same index cut-off chosen for PPV=0.70.

*k* = P * sensitivity / 0.70 (4)

Separately, we looked for the minimum AUC that would ensure *k* and sensitivity are at levels where the yield was at least twice as large with the device. A range of binormal distributions were simulated to look at typical sensitivities that could be obtained for a given AUC (0.65, 0.70, 0.75, 0.80, 0.85) and at the threshold where PPV equalled 0.70. At an AUC of 0.70, we found that sensitivity values were expected around 0.45.

**Clinical utility scenario**

The table below summarises the parameters that guided the evaluation of clinical utility. Key characteristics of this estimand are:

- the assumption that clinicians have adequate resourcing to review all antibiotics above a classifier cut-off corresponding to a PPV of 0.70. In the median hospital presented in Figure 1 of the manuscript, this is 64% of the 55 patients needing a change of therapy (*k*=35).
- a like-for-like number of patients reviewed with or without the device (equal staff resourcing with or without the device).
- the assumption that the prevalence of inappropriate prescribing is close to 30% (since PPV is a function of this prevalence).

|  | **With the device** | **Without the device** |
| --- | --- | --- |
| Minimum sensitivity requirement at PPV=0.70 | 0.45 | N/A |
| Number of patients to review | *k =* P * 0.45 / 0.70 ~ 0.64 P | Set to equal number |
| Yield | *Y_1_* = sensitivity * P  = 0.45 P | *Y_0_* = *k* * 0.3 ~ 0.19 P  (0.3 prevalence of inappropriate prescriptions) |
| Additional yield | 2.33 times greater |  |

# Subgroup performance by ethnic group

## Ethnicity subgroup cross-validation performance and training dataset sample sizes

| **Ethnic group** | **N** | **Cross-validation AUC  (95% confidence interval)** |
| --- | --- | --- |
| White | 1,379 | 0.73 (0.66, 0.80) |
| Mixed | 43 | 0.77 (0.33, 1.00) |
| Asian | 196 | 0.76 (0.56, 0.88) |
| Black | 195 | 0.79 (0.62, 0.95) |
| Other | 267 | 0.71 (0.54, 0.88) |
| Unknown | 547 | 0.76 (0.64, 0.87) |

# References

1. Dutey-Magni PF, Cawthorn A. snomedizer: R Interface to the SNOMED CT Terminology Server REST API. 2021. doi: 10.5281/zenodo.5705568

2. European Committee on Antimicrobial Susceptibility Testing. EUCAST Expert Rules: Intrinsic Resistance and Unusual Phenotypes version 3.3. (4 January 2022). European Society of Clinical Microbiology and Infectious Diseases; 2022. Available from: https://www.eucast.org/fileadmin/src/media/PDFs/EUCAST_files/Expert_Rules/2021/Intrinsic_Resistance_and_Unusual_Phenotypes_Tables_v3.3_20220104.pdf [accessed Feb 2, 2022]

3. Berends MS, Luz CF, Friedrich AW, Sinha BNM, Albers CJ, Glasner C. AMR – An R Package for Working with Antimicrobial Resistance Data. bioRxiv 2019 Jan 1;810622. doi: 10.1101/810622

4. Rawson TM, Charani E, Moore LSP, Hernandez B, Castro-Sánchez E, Herrero P, Georgiou P, Holmes AH. Mapping the decision pathways of acute infection management in secondary care among UK medical physicians: A qualitative study. BMC Med BMC Medicine; 2016;14(1):1–10. PMID:27938372

5. Seaton RA, Nathwani D, Burton P, McLaughlin C, MacKenzie AR, Dundas S, Ziglam H, Gourlay Y, Beard K, Douglas E. Point prevalence survey of antibiotic use in Scottish hospitals utilising the Glasgow Antimicrobial Audit Tool (GAAT). Int J Antimicrob Agents 2007 Jun;29(6):693–699. doi: 10.1016/j.ijantimicag.2006.10.020

6. Akhloufi H, Hulscher M, Melles DC, Prins JM, van der Sijs H, Verbon A. Development of operationalized intravenous to oral antibiotic switch criteria. J Antimicrob Chemother 2017;72(2):543–546. PMID:27999021

7. Lim WS. Defining community acquired pneumonia severity on presentation to hospital: an international derivation and validation study. Thorax 2003 May 1;58(5):377–382. doi: 10.1136/thorax.58.5.377

8. Nanda N, Juthani-Mehta M. Novel Biomarkers for the Diagnosis of Urinary Tract Infection–-A systematic Review. Biomark Insights 2009;4:BMI.S3155. PMID:19707519

9. Dutey-Magni PF, Shallcross L. Ramses: R package for Antimicrobial Stewardship & Surveillance. 2022. Available from: https://ramses-antibiotics.web.app/

10. Berends MS, Luz CF, Friedrich AW, Sinha BNM, Albers CJ, Glasner C. AMR: Antimicrobial Resistance Analysis. 2022. Available from: https://cran.r-project.org/package=AMR

11. Quan H, Sundararajan V, Halfon P, Fong A, Burnand B, Luthi J-C, Saunders LD, Beck CA, Feasby TE, Ghali WA. Coding Algorithms for Defining Comorbidities in ICD-9-CM and ICD-10 Administrative Data. Med Care 2005 Nov;43(11):1130–1139. doi: 10.1097/01.mlr.0000182534.19832.83

12. Magiorakos A-P, Srinivasan A, Carey RB, Carmeli Y, Falagas ME, Giske CG, Harbarth S, Hindler JF, Kahlmeter G, Olsson-Liljequist B, Paterson DL, Rice LB, Stelling J, Struelens MJ, Vatopoulos A, Weber JT, Monnet DL. Multidrug-resistant, extensively drug-resistant and pandrug-resistant bacteria: an international expert proposal for interim standard definitions for acquired resistance. Clin Microbiol Infect European Society of Clinical Infectious Diseases; 2012 Mar;18(3):268–281. doi: 10.1111/j.1469-0691.2011.03570.x
